# Inhibition of CREB-CBP Signaling Improves Fibroblast Plasticity for Direct Cardiac Reprogramming

**DOI:** 10.3390/cells10071572

**Published:** 2021-06-22

**Authors:** Emre Bektik, Yu Sun, Adrienne T. Dennis, Phraew Sakon, Dandan Yang, Isabelle Deschênes, Ji-Dong Fu

**Affiliations:** 1 Department of Physiology, Cell Biology College of Medicine, Ohio State University, 333 W 10th Avenue, Columbus, OH 43210, USA; Emre.Bektik@childrens.harvard.edu (E.B.); dandan.yang@osumc.edu (D.Y.); Isabelle.Deschenes@osumc.edu (I.D.); 2 Boston Children’s Hospital, Harvard Medical School, 300 Longwood Avenue, Boston, MA 02115, USA; psakon@syr.edu; 3 Heart and Vascular Research Center, Department of Medicine, MetroHealth Campus, Case Western Reserve University, Cleveland, OH 44109, USA; suny4@ccf.org (Y.S.); adennis@metrohealth.org (A.T.D.)

**Keywords:** induced cardiomyocyte, epigenetic reprogramming, heart regeneration, cell plasticity, trans-differentiation, cAMP, PKA, CREB-CBP

## Abstract

Direct cardiac reprogramming of fibroblasts into induced cardiomyocytes (iCMs) is a promising approach but remains a challenge in heart regeneration. Efforts have focused on improving the efficiency by understanding fundamental mechanisms. One major challenge is that the plasticity of cultured fibroblast varies batch to batch with unknown mechanisms. Here, we noticed a portion of in vitro cultured fibroblasts have been activated to differentiate into myofibroblasts, marked by the expression of αSMA, even in primary cell cultures. Both forskolin, which increases cAMP levels, and TGFβ inhibitor SB431542 can efficiently suppress myofibroblast differentiation of cultured fibroblasts. However, SB431542 improved but forskolin blocked iCM reprogramming of fibroblasts that were infected with retroviruses of Gata4, Mef2c, and Tbx5 (GMT). Moreover, inhibitors of cAMP downstream signaling pathways, PKA or CREB-CBP, significantly improved the efficiency of reprogramming. Consistently, inhibition of p38/MAPK, another upstream regulator of CREB-CBP, also improved reprogramming efficiency. We then investigated if inhibition of these signaling pathways in primary cultured fibroblasts could improve their plasticity for reprogramming and found that preconditioning of cultured fibroblasts with CREB-CBP inhibitor significantly improved the cellular plasticity of fibroblasts to be reprogrammed, yielding ~2-fold more iCMs than untreated control cells. In conclusion, suppression of CREB-CBP signaling improves fibroblast plasticity for direct cardiac reprogramming.

## 1. Introduction

Heart disease is the leading cause of global mortality. The most common form of heart disease is ischemic heart disease, in which healthy myocardium lacks oxygen and nutrient supplies and as a result undergoes apoptosis. Dead cardiomyocytes (CMs) are replaced by fibrotic scar tissue, which is generated by activated fibroblasts/myofibroblasts residing within the heart [1]. Adult mammalian cardiomyocytes have limited capacity to regenerate following an injury in heart tissue; therefore, an ischemic heart requires an effective replacement therapy to recover heart function [2,3]. Recent studies found that mouse [4,5,6,7] and human fibroblasts [8,9,10,11] could be directly reprogrammed into functional induced-CMs (iCMs), which offers a potential therapeutic approach to prevent scar formation and replace dead myocardium.

Since the proof-of-concept discovery of direct cardiac reprogramming [4], many studies have focused on improving the reprogramming efficiency through investigating molecular and epigenetic mechanisms of reprogramming. Various strategies, including optimized gene delivery methods [12,13], inhibition of epigenetic barriers [14,15], and pro-fibrotic signaling [16,17,18], manipulation of cell-cycle in fibroblasts [14,19], and optimization of iCM cell culture [20,21], have been investigated to enhance the reprogramming efficiency. The majority of studies so far have focused on reprogramming mechanisms after initiation of reprogramming; however, it has been known that the fibroblast state prior to reprogramming induction is also critical for a success of efficient reprogramming in vitro. Fibroblasts in the heart can be classified into four major cellular states: quiescent fibroblast, activated fibroblast, myofibroblast, and matrifibrocyte, each showing different plasticity with their special epigenetic inheritance [22,23]. A portion of fibroblasts showed resistance to epigenetic reprogramming [12,24] and varying degrees of reprogramming have been also noticed in iCMs undergoing reprogramming [11,24,25], which might be due to the heterogeneous states of isolated fibroblasts. Pretreatments of in vitro cultured fibroblasts before initiation of reprogramming has a significant impact on the reprogramming progress of fibroblasts. For example, mitomycin-C treatment that permanently inactivates the proliferation of fibroblasts decreased the yield of reprogrammed iCMs [15], while transiently cell-cycle synchronized fibroblasts have improved plasticity for reprogramming [19,25]. TGFβ is the most potent inducer of myofibroblasts differentiation [26]; it has been found that suppression of the fibrotic TGFβ signaling pathway during the reprogramming significantly increased the efficiency of iCM reprogramming [18,27], suggesting that manipulation of fibroblast state (e.g., myofibroblast differentiation) plays a significant role in improving fibroblast plasticity for reprogramming [28]. However, it has not been studied whether myofibroblast differentiation of in vitro cultured fibroblasts is associated with the plasticity of fibroblasts to be reprogrammed. It has been reported that TGFβ and cAMP signaling pathways are involved in regulation of myofibroblast differentiation [29,30]. Therefore, we were curious to investigate the role of cAMP and its downstream signaling in iCM reprogramming and explored the molecular mechanism that influences the fibroblast plasticity in epigenetic reprogramming in vitro.

In this study, we noticed that myofibroblast differentiation is generally activated in cultured fibroblasts, including neonatal mouse tail-tip fibroblasts (TTFs) and mouse embryonic fibroblasts (MEFs), which might be associated with the varying efficiency of iCM reprogramming. We studied the roles of TGFβ and cAMP signaling pathways in myofibroblast differentiation and iCM reprogramming of TTFs and MEFs. We next investigated the actions of chemical compounds, which inhibit p38/MAPK or the cAMP signaling downstream molecules (PKA and CREB-CBP), on iCM reprogramming and their role in regulation of the fibroblast plasticity, with a goal to better understand cellular signaling pathways that promote plasticity of mouse fibroblasts for reprogramming induction.

## 2. Materials and Methods

### 2.1. Transgenic Mouse Line

We used transgenic αMHC-GFP mice that were previously generated [4]. Animal experiments were approved by the Institutional Animal Care and Use Committee (IACUC) at Case Western Reserve University (2015-0058) and the Ohio State University (2019A00000085), and all animals were handled according to the university guidelines.

### 2.2. Neonatal Tail-Tip Fibroblast Isolation

Neonatal mouse tail-tip fibroblasts (TTFs) were prepared by explant-culture methods [31]. Briefly, tail-tips were harvested from neonatal αMHC-GFP transgenic mice (P0.5) and minced into small pieces (<1 mm^3^). The minced tissues were plated on gelatin-coated dishes for 5–7 days in fibroblast media (DMEM with 10% FBS [Hyclone, Logan, UT, USA; Fisher Scientific, Pittsburgh, PA, USA], NEAA (Gibco, Gaithersburg, MD, USA), and L-glutamine [Gibco, Gaithersburg, MD, USA]). Explanted TTFs were digested into single cells, filtered through 40 µm cell strainer (Falcon, ThermoFisher Sci., Tewksbury, MA, USA), and used for iCM reprogramming.

### 2.3. Mouse Embryonic Fibroblast Isolation

Mouse embryonic fibroblasts were isolated as published [19]. Briefly, MEFs were extracted from αMHC-GFP transgenic embryos (E13.5–4.5) in 0.125% Trypsin/EDTA, filtered through a 40 µm cell strainer, and cultured in fibroblast media until the cultured cells became confluent. Cells were then either stored at −80 °C or freshly passaged for reprogramming.

For the pre-treatment experiments, TGFβ inhibitor SB431542 (1 µmol/L, Cat#13031, Cayman Chem. Ann Arbor, MI, USA), p38/MAPK inhibitor SB203580 (100 nmol/L, Cat#1202, Tocris, Minneapolis, MN, USA), or 2.5 µmol/L CREB-CBP interaction inhibitor (Cat#217505, Millipore Sigma, Burlington, MA, USA) were added into the culture media 24 h post-culture of freshly isolated MEFs. Cells were treated with compounds all the time, including culture and frozen media, until they are infected with GMT retroviruses for reprogramming.

### 2.4. Direct Reprogramming and Flow Cytometry

Retroviruses (RV) were generated for reprogramming factors as previously reported [4,19]. Briefly, pMX retroviral plasmids for individual transcription factors (GMT: Gata4, Mef2c, and Tbx5) were transfected into 90% confluent Platinum E cells (Cell Biolabs, San Diego, CA, USA) with FugeneHD (Promega) as suggested by manufacturer’s protocol. Transfection media was removed the next day and replaced with fresh PlatE media (DMEM with 10% FBS). Approximately 48 h after transfection, culture media containing viruses were harvested and filtered with 0.45 µM filters (Nalgene, ThermoFisher Sci., Waltham, MA, USA) and used freshly for iCM reprogramming. A control RV of dsRed was produced in each batch of virus generation and served as the negative control of iCM reprograming.

Fibroblasts were seeded at a density of 120,000 cells per well in a 6-well plate one day before retrovirus infection and then infected with a mixture of freshly-made GMT viruses (0.5 mL of each) with 8 µg/mL polybrene (Millipore, Cleveland, OH, USA) supplement. Next day, retroviral media was removed and replaced with fresh iCM media (DMEM/M199 [4:1] with 10% heat-inactivated FBS, NEAA, and L-glutamine) with or without chemical compounds; forskolin (10 µmol/L, Cat#6886, Sigma, Saint Louis, MO, USA), TGFβ inhibitor SB431542 (1 µmol/L), p38/MAPK inhibitor SB203580 (100 nmol/L), CREB inhibitor (2.5 µmol/L), or PKA inhibitor H89 dihydrochloride (1 µmol/L, Cat# 2910, Tocris, Minneapolis, MN, USA), or PKI 14-22 amide (peptide inhibitor of PKA; 5 µmol/L, Cat#2546, Tocris) for 7 days. Reprogrammed fibroblasts were maintained in iCM media with media change every 2 to 3 days. To evaluate the outcome of reprogramming, iCMs were digested into single cells with 0.05% Trypsin/EDTA and resuspended in FACS buffer (5% FBS and 2 mM EDTA in 1X PBS). BD Accuri C6 flow cytometer (BD Biosciences) was used to evaluate percentage and absolute number of αMHC-GFP^+^ iCMs. RV-dsRed infected fibroblasts were used as the negative control to set up the gating of αMHC-GFP^+^ population in FACS analysis; αMHC-GFP^+^ iCMs at Day 3 post-GMT induction was defined as GFP^low^ population and used to set up the gating of GFP^high^ population.

### 2.5. Quantitative Real-Time PCR (qRT-PCR)

To evaluate expression of cardiac markers in iCMs, reprogramming cells of entire cell population were harvested at Day 7–8 post-reprogramming and lysed in Trizol (Cat#15596018, ThermoFisher Sci., Waltham, MA, USA), and total RNA was extracted as per manufacturer’s protocol. cDNA was generated from 2 µg RNA samples using MultiScribe ™ reverse transcription kit (Cat#4311235, ThermoFisher Sci.). Quantitative PCR assays were performed with SsoFast™ EvaGreen^®^ supermix (Cat#1725201, BioRad, Hercules, CA, USA) by a 7300 Real-Time PCR system (Applied Biosystems, Foster City, CA, USA). The primers of cardiac markers are *Myh6* (F: GCCCAGTACCTCCGAAAGTC; R: GCCTTAACATACTCCTCCTTGTC), *Ryr2* (F: ACGGCGACCATCCACAAAG; R: AAAGTCTGTTGCCAAATCCTTCT), *Tnnt2* (F: ACAGAGGAGGCCAACGTAGA; R: AAGTTGGGCATGAAGAGCCT), and *Actc1* (F: TGCCATGTATGTCGCCATCC; R: CACCATCGCCAGAATCCAGA). The primers of myofibroblast markers include *Acta2* (F: ATCACCAACTGGGACGACAT; R: CATACATGGCTGGGACATTG), *Pten* (F: TGGATTCGACTTAGACTTGACCT; R: GCGGTGTCATAATGTCTCTCAG), *Pxn* (F: CAAACGGCCAGTGTTCTTGTC; R: TGTGTGGTTTCCAGTTGGGTA), and *Vcl* (F: TGGACGGCAAAGCCATTCC; R: GCTGGTGGCATATCTCTCTTCAG). The expression of genes was quantified and normalized to a housekeeping gene *Gapdh* (F: AGGTCGGTGTGAACGGATTTG; R: TGTAGACCATGTAGTTGAGGTCA). Data was shown as a fold change of gene expression compared to GMT control group.

### 2.6. Assays of Myofibroblast Differentiation

The expression of myofibroblast markers in cultured fibroblasts was evaluated by qRT-PCR and immunostaining. To suppress the differentiation of myofibroblasts, fibroblasts were treated with 1 µmol/L SB431542 or 10 µmol/L forskolin for 48 h. Cultured cells were harvested and lysed in Trizol, and the mRNA expression of myofibroblast markers was evaluated by qRT-PCR as described above.

For immunostaining of a myofibroblast marker αSMA and a fibroblast marker Thy1, cultured fibroblasts were fixed with 4% PFA and incubated with primary antibodies of mouse anti-αSMA (1:500, Cat.# MA5–11547, ThermoFisher Sci., Waltham, MA, USA) and APC-conjugated rat anti-Thy1.2 (1:200, Cat.# 13-0903-81, ThermoFisher Scientific) at 4 °C overnight, and then incubated with secondary antibodies of AlexaFluor-488 goat anti-mouse IgG (1:400, Cat#11029, Invitrogen, Waltham, MA, USA). The stained samples were imaged by DMi8 Leica fluorescent microscope (Leica Microsystems, Buffalo Grove, IL, USA) and APC-fluorescence signals were exhibited as pseudo-red color in images.

### 2.7. Statistical Analyses

All data were expressed as mean ± SEM. For statistical analysis, all experimental groups included at least three biological replicates, and the statistical significance was examined by two-tailed paired Student’s *t*-test or first one-way ANOVA and subsequent contrasts with multiple comparison correction. *p*-Values of < 0.05 were accepted as statistically significant. * *p* < 0.05, ** *p* < 0.01, *** *p* < 0.001; # *p* < 0.05, ## *p* < 0.01, ### *p* < 0.001.

## 3. Results

### 3.1. In Vitro Cultured Fibroblasts Contain Differentiated Myofibroblasts Prior to the Induction of Reprogramming

**Figure 1:**
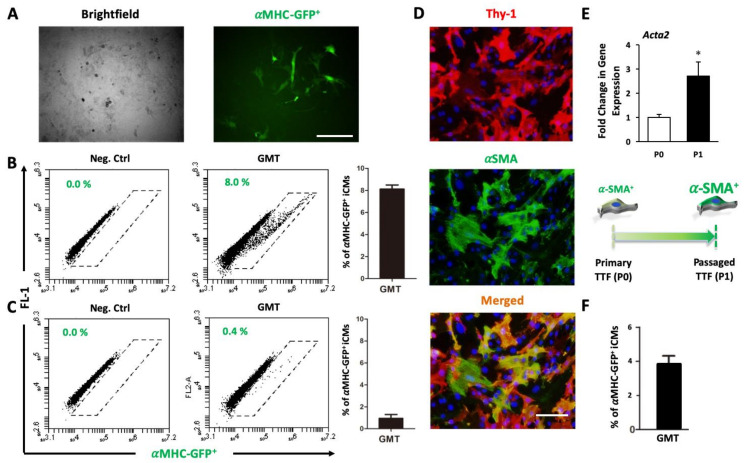
The plasticity of in vitro cultured fibroblasts varies. (**A**) Brightfield (left) and GFP (right) representative images of induced cardiomyocyte (iCM) reprogramming (Day 8) from tail-tip fibroblasts (TTFs). Bars indicate 50 μm. (**B**–**C**) Representative FACS plots and average percentage of αMHC-GFP^+^ iCMs reprogrammed from TTFs with relatively good (**B**) and low (**C**) efficiency. (**D**) Immunostaining of TTFs for fibroblast marker Thy1 (in red) and myofibroblast marker αSMA (in green), showing that cultured fibroblasts were differentiated into myofibroblasts. Bars indicate 200 μm. (**E**) qRT-PCR shows that gene expression of *Acta2*, a myofibroblast marker, was significantly higher in single-passaged cells TTFs (P1) than that in primary TTFs (P0), indicating myofibroblast differentiation in cultured fibroblasts. (**F**) Average percentage of αMHC-GFP^+^ iCMs reprogrammed from passaged TTFs. * *p* < 0.05 vs. P0 fibroblasts.

Although proper expression of reprogramming factors (e.g., GMT: Gata4, Mef2c, and Tbx5) in fibroblasts is essential to epigenetic reprogramming, the quality or status of fibroblasts prior to the induction of reprogramming is an important factor for successful and efficient reprogramming. We noticed that some batches of reprogrammed neonatal tail-tip fibroblasts (TTFs) had relatively good efficiency (8.46 ± 0.64%, n = 5, Figure 1A,B) while some had very poor efficiency or failed to be reprogrammed (0.78 ± 0.34%, n = 5, Figure 1C). Although many factors should be considered, we asked if it is associated with myofibroblast differentiation, which has been observed in in vitro cultured fibroblasts with increased expression of fibrotic genes [32,33]. Indeed, our immunostaining did show that the myofibroblast marker alpha smooth muscle actin (αSMA) was expressed in many primary cultured TTFs (Figure 1D), and the expression of the αSMA gene (*Acta2*) significantly increased in secondary-cultured TTFs (2.72 ± 0.58 folds, n = 4, *p* = 0.0117) even after only one passage of primary cultured fibroblasts (Figure 1E), which eventually reduced reprogramming efficiency of passaged TTFs to relatively low levels (~4%, n = 3, Figure 1F). It has been reported in many laboratories that freshly-isolated fibroblasts, rather than passaged fibroblasts, are recommended to be used for a good efficiency of reprogramming [1,3,34]; therefore, we speculated that passaged fibroblasts lose plasticity for reprogramming via alternating fibroblast state, such as differentiating into myofibroblasts.

**Figure 2:**
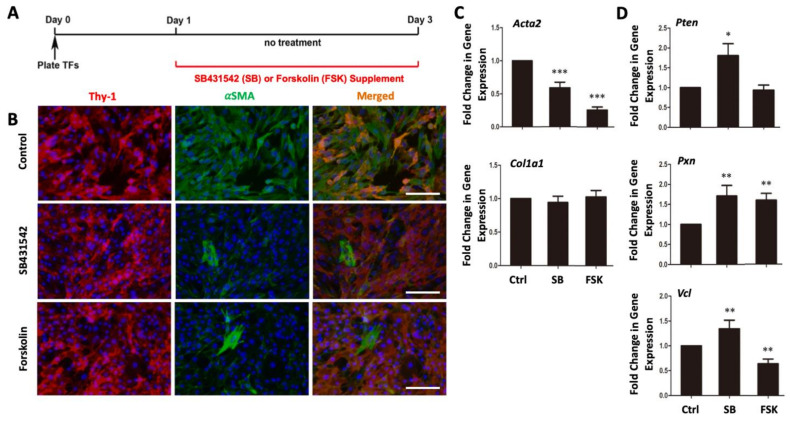
Inhibition of TGFβ or activation of cAMP signaling pathways suppress myofibroblast differentiation of cultured fibroblasts. (**A**) Experimental design of compound treatments. (**B**) Immunostaining of Thy1 (a fibroblast marker; in red) and αSMA (a myofibroblast marker; in green) shows that the myofibroblast differentiation was inhibited by TGFβ inhibitor SB431542 (SB) or forskolin (FSK). Bars indicate 100 μm. (**C**,**D**) Fold changes of the mRNA expression of myofibroblast differentiation markers were significantly decreased in SB- or FSK-treated TTFs compared to untreated control (Ctrl) cells. * *p* < 0.05, ** *p* < 0.01, *** *p* < 0.001 vs. Ctrl.

To prevent myofibroblast differentiation of cultured cells, we treated fresly-isolated TTFs with a TGFβ inhibitor (SB431542) or a cAMP activator (forskolin) (Figure 2A) for 48 h. We found that both compounds affectively suppressed myofibroblast differentiation, indicated by the significantly-decreased expression of αSMA in Thy1^+^ fibroblast population (Figure 2B). Our qRT-PCR confirmed that the expression of myofibroblast late-differentiation marker αSMA was significantly suppressed by both SB431542 and forskolin (Figure 2C). Interestingly, SB431524 increased the expression of proto-myofibroblast markers, including Pten (phosphatase and tensin homolog), Pxn (paxillin), and Vcl (vinculin), which are expressed in early stages of myofibroblast differentiation and whose expression levels keep rising in prolonged cell cultures [33,35]; while forskolin indeed suppressed the expression of Vcl (Figure 2D). These results suggested that full myofibroblast differentiation of cultured fibroblasts can be efficiently suppressed by inhibiting TGFβ signaling or activating cAMP signaling; however, inhibition of the TGFβ signaling pathway seems to promote early differentiation but inhibit the full differentiation of myofibroblasts while activation of cAMP signaling plays varied actions and inhibits full differentiation of myofibroblasts.

### 3.2. Post-Induction Treatments of SB431542 and Forskolin Have Opposite Effects on iCM Reprogramming

**Figure 3:**
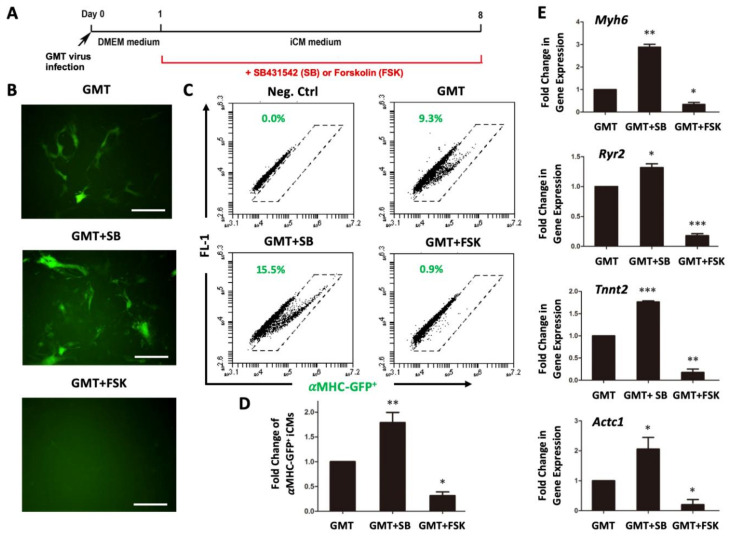
Post-induction treatments of SB431542 (SB) and forskolin (FSK) have opposite effect on iCM reprogramming of tail-tip fibroblasts (TTF). (**A**) Experimental strategy of post-induction treatment during iCM reprogramming. (**B**–**C**) Representative fluorescent images (**B**) and FACS plots (**C**) of iCM reprogramming at Day 8 after infection. Bars indicate 50 μm. (**D**) Average fold changes of reprogrammed αMHC-GFP^+^ iCMs by SB431542 and FSK. (**E**) Fold changes of cardiac gene expression in reprogrammed fibroblasts, including *Myh6*, *Ryr2*, *Tnnt2*, and *Actc1*. * *p* < 0.05, ** *p* < 0.01, or *** *p* < 0.001 vs. GMT ctrl.

**Figure 4:**
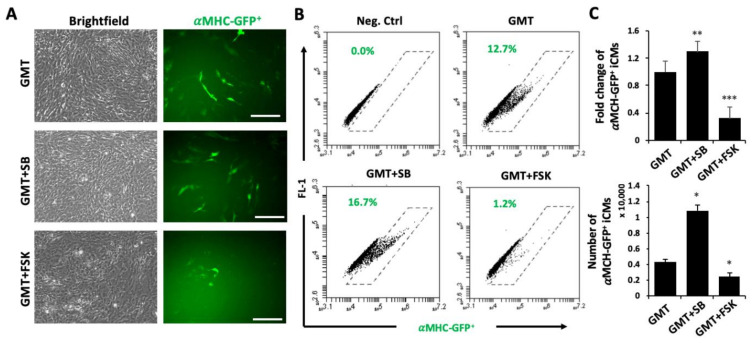
Forskolin inhibits iCM reprogramming of MEFs. (**A**–**B**) Representative brightfield (upper lane) and fluorescent (lower lane) images (**A**) and FACS plots (**B**) of αMHC-GFP^+^ iCMs at Day8 of reprogramming with post-induction treatments of SB431542 (SB) or forskolin (FSK). Bars indicate 50 μm. (**C**) Average fold changes of GMT-reprogrammed αMHC-GFP^+^ iCMs by SB (1.3 ± 0.14%, n = 4, *p* = 0.009) or FSK (0.32 ± 0.17%, n = 4, *p* < 0.001). Average yield of αMHC-GFP^+^ iCMs by GMT (4302 ± 377 cells; n = 3) was increased to 10,851 ± 759 cells (n = 3, *p* = 0.038) by SB but decreased to 2452 ± 507 cells (n = 3, *p* = 0.016) by FSK. * *p* < 0.05 or ** *p* < 0.01 or *** *p* < 0.005 vs. GMT ctrl.

Next, we treated reprogrammed TTFs with SB431542 or forskolin for 7 days after infection of GMT retroviruses (Figure 3A) and studied the efficiency of reprogrammed αMHC-GFP^+^ iCMs. Consistent with previous reports [17,18], inhibition of TGFβ signaling by SB431542 significantly improved the reprogramming efficiency (1.80 ± 0.20 folds, n = 4, *p* = 0.029) of TTFs (Figure 3B–D). Surprisingly, activation of cAMP signaling by forskolin dramatically decreased the yield of reprogrammed αMHC-GFP^+^ cells (0.31 ± 0.17 folds, n = 4, *p* = 0.024) from TTFs (Figure 3B–D). The activation of cardiac muscle genes by GMT, including *Myh6*, *Ryr2*, *Tnnt2* and *Actc1*, were significantly inhibited by forskolin, while SB431542 facilitated their activations (Figure 3E). The inhibition of iCM reprogramming by forskolin was also observed in mouse embryonic fibroblasts (MEFs). Activation of cAMP signaling by forskolin significantly suppress iCM reprogramming of MEFs and yielded much less αMHC-GFP^+^ iCMs (Figure 4). Our results suggested that activation of cAMP signaling pathway might be a barrier in the epigenetic reprogramming of iCMs.

### 3.3. Inhibition of cAMP Downstream PKA Signaling Post-GMT Induction Improves Reprogramming

**Figure 5:**
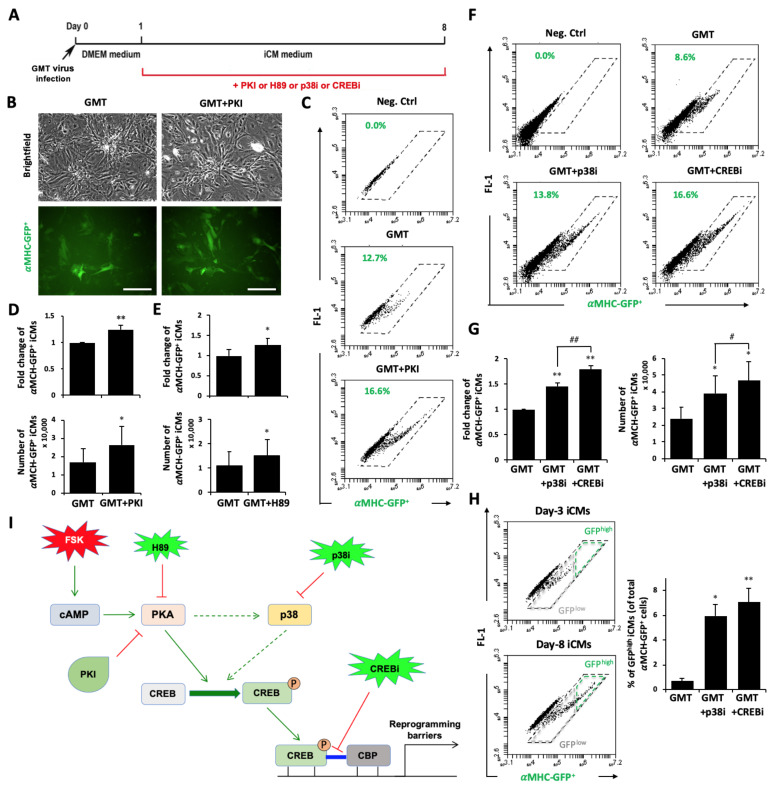
Inhibition of PKA signaling pathway improves iCM reprogramming of MEFs. (**A**) Experimental design of reprogramming with post-GMT inhibition of cAMP downstream targets. (**B**,**C**) Representative brightfield and GFP images (**B**) and FACS plots (**C**) of αMHC-GFP^+^ iCMs treated with or w/o a PKA peptide inhibitor (PKI). Bars indicate 50 μm. (**D**) Bar graphs show fold change of percentage (left panel) and absolute number (right panel) of αMHC-GFP^+^ iCMs in reprogramming of GMT or GMT+PKI. (**E**) H89 dichloride, a chemical inhibitor of Protein kinase A, increased the percentage (upper panel) and absolute number (lower panel) of αMHC-GFP^+^ iCMs. (**F**) Representative FACS plots of GMT reprogramming with or w/o a chemical inhibitor of p38 (p38i) SB203580 or a CREB inhibitor (CREBi). (**G**) Average fold changes of the percentage (upper panel) and the absolute number (lower panel) of reprogrammed αMHC-GFP^+^ iCMs by p38i or CREBi. (**H**) Yield of αMHC-GFP^high^ iCMs with post-GMT treatment of p38i or CREBi. Representative FACS plots show the gating for GFP^low^ vs. GFP^high^ cell populations among total αMHC-GFP^+^ iCMs. Bar graph shows average percentage of GFP^high^ iCMs with or without compounds. (**I**) Diagram showing cAMP/PKA/CREB signaling pathway activated by forskolin. * *p* < 0.05 or ** *p* < 0.01 vs. GMT ctrl; # *p* < 0.05, ## *p* < 0.01 vs. GMT+p38i.

Forskolin activates critical signaling pathways via a secondary messenger, cAMP [36,37], which activates protein kinase A (PKA) [38] and results in phosphorylation of numerous targets, including transcriptional regulators (e.g., CREB [39]), that mediate critical biological processes. Therefore, we asked if the PKA signaling pathway mediates the observed inhibition of iCM reprogramming by forskolin-induced cAMP. We studied a post-induction treatment of PKI 14-22 amide, a specific peptide that binds on catalytic site of PKA and inhibits its kinase activity, and found that PKI significantly improved the reprogramming efficiency (1.25 ± 0.08 folds, n = 6, *p* = 0.010; Figure 5A–D) and increased the number of reprogrammed αMHC-GFP^+^ iCMs (18,352 ± 7123 vs. 12,707 ± 4741 of the GMT control group; n = 8, *p* = 0.042; Figure 5D). The enhancement of iCM reprogramming by PKI was also observed in TTFs (data not shown). We had also applied H89 dihydrochloride, a chemical inhibitor of PKA, post-GMT induction and observed a consistent enhancement of reprogramming with increased percentage (1.27 ± 0.17, n = 7, *p* = 0.028) and absolute number (14,989 ± 6588 vs. 11,062 ± 5588 of the GMT control group; n = 7, *p* = 0.0276) of reprogrammed αMHC-GFP^+^ iCMs (Figure 5E).

We next investigated CREB, a downstream target of PKA signaling pathway, and p38/MAPK pathway, which is known to interact with both PKA and CREB pathways [39,40]. We found that a chemical inhibitor of p38/MAPK signaling (p38i), SB203580, could significantly enhance the efficiency (1.46 ± 0.05 folds; n = 7, *p* < 0.001) of iCM reprogramming and increased the number of reprogrammed αMHC-GFP^+^ iCMs from 24,044 ± 6578 cells in the GMT control group to 39,062 ± 10,401 (n = 7, *p* = 0.0174) (Figure 5F–G). Similarly, an inhibitor of CREB and CBP interaction improved the efficiency of reprogramming (1.79 ± 0.08 folds; n = 7, *p* < 0.001) and increased cell number (46,790 ± 11,584; n = 7, *p* = 0.010) of iCMs compared to GMT ctrl. Noticeably, CREBi was more effective than p38i at improving both efficiency (*p* = 0.008) and cell number (*p* = 0.024) of iCMs (Figure 5G). The enhancement of reprogramming by CREBi was also observed in cardiac fibroblasts (data not shown). We also investigated whether these two compounds could improve the percentage of relatively higher quality of iCMs (GFP^high^). Previously, we found that the GFP^high^ portion of iCMs possesses a higher degree of reprogramming with increased cardiac gene expression and percentage of cardiac troponin-T^+^ (cTnT^+^) iCM population [19]. We found that both p38i (4.35 ± 0.9%, n = 6, *p* = 0.0134) and CREBi (5.23 ± 1.14%, n = 6, *p* = 0.0095) significantly improved percentage of GFP^high^ iCMs compared to GMT ctrl (0.53 ± 0.26%, n = 6) (Figure 5H). Our results demonstrate that CREB, a major downstream signaling of both PKA and p38 signaling pathways, plays an important role in regulation of iCM reprogramming (Figure 5I).

### 3.4. Pretreatment of Primary Fibroblasts with CREB-CBP Inhibitor Improves Fibroblast Plasticity for Reprogramming

**Figure 6:**
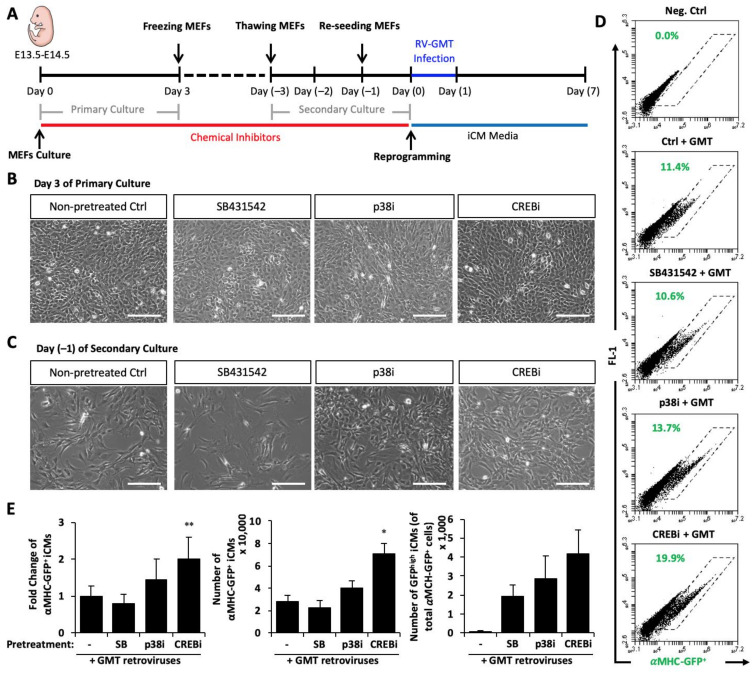
Pretreatment of MEFs with CREB-CBP inhibitor preserves cellular plasticity for reprogramming. (**A**) Experimental design of MEF culture and pretreatments of TGFβ inhibitor (SB431542), p38 inhibitor (p38i), or CREB inhibitor (CREBi) before direct cardiac reprogramming by GMT retrovirus. (**B**) Representative images of compound-treated MEFs at Day 3 of primary culture. (**C**) Representative images of MEFs at Day (−1) of secondary culture. (**D**) Representative FACS plots of αMHC-GFP^+^ iCMs reprogrammed from MEFs with or w/o pretreatments of chemical compounds. (**E**) Average fold changes of the percentage (left panel) and absolute number (middle panel) of αMHC-GFP^+^ iCMs, and average number of GFP^high^ population among total αMHC-GFP^+^ iCMs (right panel) from MEFs pretreated with SB431542, p38i or CREBi. MEFs without pre-treatment of the same-batch culture were used as the ctrl group. * *p* < 0.05 or ** *p* < 0.01 vs. non-pretreated ctrl. Bars indicate 50 μm.

We further asked whether any of these signaling pathways could maintain the plasticity of in vitro cultured fibroblasts to be reprogrammed. We treated fibroblasts with TGFβ inhibitor (TGFβi), p38i, or CREBi from the initial time of primary cultures until MEFs were used for GMT-induction (Figure 6A). We did not notice obvious morphology changes of primary cultured cells among groups with or without chemical inhibitors. Primary cultures of MEFs were proliferating healthily with high cell density in all groups (Figure 6B). We found that chemical inhibitors did have a different effect on the proliferation capability of MEFs after freezing and passing of secondary cell culture; p38i and CREBi improved cell proliferation with much higher density of MEFs than the control group, while TGFβi suppressed the proliferation of MEFs (Figure 6C). Remarkably, CREBi-pretreated MEFs showed a significantly-enhanced plasticity to be reprogrammed by GMT, with a better reprogramming efficiency (2.02 ± 0.58 folds; n = 3, *p* = 0.0012) and an increased number of reprogrammed αMHC-GFP^+^ iCMs (71,094 ± 9311 cells vs. 28,463 ± 4963 cells in the untreated control group; n = 3, *p* = 0.048), while pretreatments of TGFβi or p38i had no significant effect on the reprogramming plasticity. Noticeably, all three compounds had marginal effect on number of GFP^high^ iCMs (TGFβi: 1913 ± 600 cells, n = 3, *p* = 0.299; p38i: 2880 ± 1188 cells, n = 3, *p* = 0.442; CREBi: 4170 ± 1273 cells, n = 3, 0.263) compared to GMT ctrl (98 ± 20 cells, n = 3), suggesting that pretreatment of CREBi rather quantitatively improves reprogramming. Overall, our study demonstrated that inhibition of CREB signaling improves the plasticity of fibroblasts for cardiac epigenetic reprogramming.

## 4. Discussion

In this study, we investigated novel signaling pathways, including TGFβ, cAMP/PKA, and p38, that are involved in direct cardiac reprogramming and found that inhibition of cAMP/PKA/CREB signaling could preserve the plasticity of cultured fibroblast to be reprogrammed.

In vitro cultured fibroblasts have different cellular states that affect the efficiency of epigenetic reprogramming, yielding a varied efficiency from batch to batch. TGFβ inhibitor SB431542 has been reported to significantly enhance iCM reprogramming [16,17,18]. The supraphysiologic matrix stiffness of the tissue culture substrate leads to liberation of latent TGFβ reservoirs from ECM and its activation at the basal level in cell culture [41,42]. Also, fetal bovine serum (FBS) used in fibroblast cultures contains some latent and active TGFβ cytokines [43,44]. Therefore, myofibroblast differentiation commonly exists in in vitro cultured fibroblasts with increased expression of fibrotic genes [32,33], and inhibition of TGFβ signaling can effectively suppress myofibroblast differentiation [45,46]. These discoveries suggest that myofibroblast status may be an epigenetic barrier for direct cardiac reprogramming and suppression of myofibroblast differentiation might be a good strategy to improve the reprogramming efficiency. In our study, cAMP signaling pathway, activated by forskolin, could suppress myofibroblast differentiation, as previously reported [29,30]. It seems a rational hypothesis that forskolin may enhance reprogramming efficiency, particularly when exclusion of forskolin from the chemical cocktail of reprogramming factors significantly decreased induction of iCMs [47]. However, in our study, forskolin blocked reprogramming induced by GMT transcription-factor cocktail, which suggests that iCM reprogramming induced by chemical or transcription-factor cocktails may differentially require cAMP signaling. Although both TGFβ inhibitor and cAMP signaling activation suppress myofibroblast differentiation, we did observe the difference that SB431542 promoted early stage of differentiation, indicated by increased expression of early markers. However, SB431542 suppressed full differentiation of myofibroblasts, indicated by decreased expression of αSMA, while forskolin could stage-independently suppress myofibroblast differentiation, which might be the underlying mechanism of how two compounds produce opposite effects on GMT-induced reprogramming of fibroblasts.

As one type of secondary messenger, cAMP has a broad range of targets in various signaling pathways, among which PKA signaling is a well-known target and is actively involved in regulation of most biological events [48]. Previously, it has been reported that inhibition of the PKA signaling pathway improves reprogramming of iPSCs [49]. In this study, inhibition of PKA by a chemical compound (H89) or specific peptide (PKI 14-22 amide) also increased the yield of iCM reprogramming. We next studied one of the well-known downstream targets of PKA signaling, CREB, which regulates fibroblast [50,51,52,53,54,55] and cardiac function and disease [56,57,58]. We found that CREB inhibitor improves iCM reprogramming and inhibition of p38 signaling, which also regulate the phosphorylation of CREB, consistently enhanced reprogramming efficiency. The p38 signaling pathway in fibroblasts could be activated by TGFβ signaling [59,60]; therefore, p38 inhibition also showed an enhancement of iCM reprogramming. These discoveries demonstrate that, post-initiation of GMT-mediated reprogramming, inhibition of cAMP/PKA/CREB signaling alters epigenetic status and enhances direct cardiac reprogramming.

We asked if these signaling pathways, which suppress myofibroblast differentiation, could modify or preserve cultured fibroblasts in a more plastic state to be reprogrammed. We pretreated primary culture of fibroblasts with different inhibitors before the induction of reprogramming and investigated their plasticity to be reprogrammed into αMHC-GFP^+^ iCMs by GMT. Neither the TGFβ inhibitor nor the p38 inhibitor improved the reprogramming efficiency of secondary-culture fibroblasts; although inhibitors of both signaling pathways have been reported to suppress myofibroblast differentiation [45,59,60]. Interestingly, CREBi-treated fibroblasts have better plasticity, and more of them were reprogrammed into αMHC-GFP^+^ iCMs than untreated control fibroblasts. The mechanism of preserved plasticity via CREB inhibition is beyond the suppression of myofibroblast differentiation. Cell-cycle synchronization facilitates the progression of iCM reprogramming [19], and inhibition of CREB signaling could suppress cell cycle in some cell lines [61,62,63]. In our study, however, we did not observe the CREB inhibitor significantly change the cell-cycle phases of cultured MEFs (data not shown); therefore, its mechanism in reprogramming is possibly not related to cell cycle. More investigations are needed to understand the mechanism of how CREB regulates plasticity of fibroblasts and how it benefits to reprogramming. Nevertheless, our discovery of preserving the reprogramming plasticity of cultured fibroblasts by inhibiting cAMP/PKA/CREB signaling provides a better mechanistic understanding of direct cardiac reprogramming in vitro.

One limitation of our study is that cardiac gene expressions were analyzed in whole population rather than in purified αMHC-GFP^+^ iCMs. Therefore, we included assays of absolute cell number of produced iCMs and percentage of GFP^high^ population among total iCMs that are more appropriate to evaluate effects of compounds on reprogramming yield. Another limitation is that the overall benefits of chemical compounds in reprogramming efficiency was limited and, in some cases, marginally affected reprogramming; it might need to suppress multiple epigenetic barriers simultaneously to achieve high efficiency of iCM reprogramming in vitro. Finally, it is possible that cAMP/PKA/CREB molecules form an axis of signaling pathway in regulation of direct cardiac reprogramming, which requires more biochemical studies of validation in future studies.
